# Disparities in Care and Outcome of Stroke Patients from Culturally and Linguistically Diverse Communities in Metropolitan Australia

**DOI:** 10.3390/jcm10245870

**Published:** 2021-12-14

**Authors:** Fatemeh Rezania, Christopher J. A. Neil, Tissa Wijeratne

**Affiliations:** 1 Department of Neurology and Stroke Service, Sunshine Hospital Western Health, St Albans, VIC 3021, Australia; rezania1982@gmail.com; 2 Department of Medicine, Melbourne Medical School University of Melbourne, Sunshine Hospital, St Albans, VIC 3021, Australia; Christopher.neil@wh.org.au; 3 Department of Public Health, La Trobe University, Bundoora, VIC 3083, Australia

**Keywords:** stroke, disparities, language barrier, clinical outcome

## Abstract

Background: Acute stroke is a time-critical emergency where diagnosis and acute management are highly dependent upon the accuracy of the patient’s history. We hypothesised that the language barrier is associated with delayed onset time to thrombolysis and poor clinical outcomes. This study aims to evaluate the effect of language barriers on time to thrombolysis and clinical outcomes in acute ischemic stroke. Concerning the method, this is a retrospective study of all patients admitted to a metropolitan stroke unit (Melbourne, Victoria, Australia) with an acute ischemic stroke treated with tissue plasminogen activator between 1/2013 and 9/2017. Baseline characteristics, thrombolysis time intervals, length of stay, discharge destination, and in-hospital mortality were compared among patients with and without a language barrier using multivariate analysis after adjustment for age, sex, stroke severity, premorbid modified Rankin Scale (mRS), and Charlson Comorbidity Index (CCI). Language barriers were defined as a primary language other than English. A total of 374 patients were included. Our findings show that 76 patients (20.3%) had a language barrier. Mean age was five years older for patients with language barriers (76.7 vs. 71.8 years, *p* = 0.004). Less non-English speaking patients had premorbid mRS score of zero (*p* = 0.002), and more had premorbid mRS score of one or two (*p* = 0.04). There was no statistically significant difference between the two groups in terms of stroke severity on presentation (*p* = 0.06). The onset to needle time was significantly longer in patients with a language barrier (188 min vs. 173 min, *p* = 0.04). Onset to arrival and door to imaging times were reassuringly similar between the two groups. However, imaging to needle time was 9 min delayed in non-English speaking patients with a marginal *p* value (65 vs. 56 min, *p* = 0.06). Patients with language barriers stayed longer in the stroke unit (six vs. four days, *p* = 0.02) and had higher discharge rates than residential aged care facilities in those admitted from home (9.2% vs. 2.3%, *p* = 0.02). In-hospital mortality was not different between the two groups (*p* = 0.8). In conclusion, language barriers were associated with almost 14 min delay in thrombolysis. The delay was primarily attributable to imaging to needle time. Language barriers were also associated with poorer clinical outcomes.

## 1. Introduction

Stroke is a leading cause of death and a substantial disability globally [1]. Approximately 80% of incident strokes are ischemic [2], where time-critical acute care is heavily dependent upon an accurate history and examination.

Rising global migration has led to cultural and linguistic diversification of communities and introduced new challenges to the healthcare system in Australia along withother immigration nations. 2016 census in Australia showed that 26% of the population was born overseas, with a 1% increase from the last census in 2011, while more than one-fifth (21%) speak a language other than English at home [3]. The Australian Stroke Clinical Registry (AuSCR) also reported that more than 7% of stroke patients in Australia speak a language other than English [4]. Little is known about the influence of language barriers on stroke care regarding time-to-thrombolysis in particular [5] and clinical outcomes such as mortality, discharge destinations, and quality of life in general [6].

This study aimed to determine the influence of language barriers on relevant thrombolysis timing intervals and clinical outcomes (mortality, discharge destination, and length of stay) within a consecutive series of stroke patients who received tissue plasminogen activator (tPA). We tested the hypothesis that the presence of a language barrier would be independently associated with delayed thrombolysis and disparity in clinical outcomes.

## 2. Methods

This was a retrospective observational study of a cohort of patients admitted to a metropolitan stroke unit of Western Health, Melbourne, Victoria, Australia, with an acute ischemic stroke and treated with tPA between January 2013 and September 2017. 

Appropriate time intervals, premorbid modified Rankin Scale (mRS), National Institutes of Health Stroke Scale (NIHSS), demographic variables, and recruitment to clinical trials for thrombolysed patients are prospectively collected by our unit stroke nurse consultant for the purpose of quality improvement. These datasheets, together with electronic medical records, were retrospectively reviewed for all eligible patients. 

Language barriers were defined as a primary language other than English as recorded in our health network administration database. These data were collected and documented by a hospital clerk at the time of admission. 

The time of code stroke call was used as arrival time for in-patient strokes. 

Charlson comorbidity index was calculated as described by Goldstein et al. [7]. The stroke and hemiplegia secondary to the incident stroke were not included. 

A number of patients were recruited to two clinical trials in hyper-acute management of ischemic stroke. 

Ethical approval was granted by the ethics committee at Western Health. 

In our institution, consent for thrombolysis is obtained verbally from the patient. When a patient lacks capacity, consent is obtained from the next of kin. 

## 3. Statistical Analysis

Continuous variables were expressed as means (standard deviations) or medians (interquartile ranges). Baseline characteristics were compared between English and non-English patients using the Chi-squared test for categorical variables, Fisher exact test for binary variables, and Mann–Whitney U test for all continuous variables aside from age, which was compared using a t-test. The absolute difference in time intervals and length of stay were determined using univariate linear regression. To determine the independent effect of a language barrier on relevant time intervals and length of stay, multivariable regression models were used, adjusted for age, sex, initial stroke severity, premorbid modified Rankin Scale (for thrombolysis times), and Charlson comorbidity index (for the length of stay). 

Logistic regression models adjusted for age, sex, initial stroke severity and Charlson comorbidity index were also built to examine the effect of non-English status on in-hospital mortality and discharge destination from stroke ward.

GraphPad Prism version 7.03 (San Diego, CA, USA) was applied to analyse the baseline characteristics, and regressions were modelled using Stata version 14. *p* < 0.05 was considered statistically significant. *p*-values of regression models were calculated with bootstrap with 5000 replications.

## 4. Results

A total of 374 patients were included after excluding 22 patients due to stroke mimics (*n* = 18) and incomplete medical records (*n* = 13). English was documented as the primary language in 298 patients, while 76 patients reported a primary language other than English (79.7% vs. 20.3%). The baseline characteristics of patients are listed in Table 1. Mean age was five years older for patients with language barriers (76.7 vs. 71.8 years, *p* = 0.003). There was a similar proportion of males in the two groups (57.7% vs. 52.6%, *p* = 0.43). Less non-English speaking patients had premorbid mRS score of zero (46.6% vs. 65.7%, *p* = 0.002), and more had premorbid mRS score of one or two (18.6% vs. 9.7%, *p* = 0.04; 16% vs. 7.3%, *p* = 0.04, respectively). A comparable number of patients had mRS > 2. Stroke severity on presentation (median initial NIHSS) was slightly higher in the non-English speaking cohort; however, this was not statistically significant (9 vs. 8, *p* = 0.06). The prevalence of most common comorbidities (hypertension and hyperlipidaemia) were not different between patients with or without a language barrier (*p* = 0.16 and *p* = 0.06, respectively). CCI and other baseline comorbidities, including the history of previous stroke/TIA, were not significantly different. Recruitment to clinical trials in hyper-acute management of ischemic stroke was comparable between the two groups of patients. 

The onset to needle time was significantly longer in patients with language barriers (Adjusted difference 13.7 min, 95% CI 0.12–27.4, *p* = 0.04) (Table 2). Onset to arrival and door to imaging times were similar between English and non-English speaking (*p* = 0.81 and *p* = 0.99, respectively). However, imaging to needle time was 8.9 min delayed in patients with language barriers with a marginal *p* value (95% CI −0.57–18.5, *p* = 0.06) (Table 2). 

The in-hospital mortality rate was similar between the two groups (*p* = 0.8) (Table 3). An equal proportion of patients from each group were discharged to home or rehabilitation units from the stroke ward (*p* = 0.9 and *p* = 0.63, respectively). Clot retrieval rate was also comparable (*p* = 0.83). However, patients with a language barrier had a longer median length of stay at the stroke unit (3 days longer, 95% CI 0.35–5.8; *p* = 0.02) and higher rates of discharge to a residential aged care facility (RACF) in those admitted from home (9.2% vs. 2.3%, adjusted OR 3.6, 95% CI 1.1–11.4, *p* = 0.02) (Table 3).

## 5. Discussion

The presence of a language barrier was associated with approximately 14 min delay in onset to needle time after adjusting for baseline characteristics. The observed delay was mainly attributable to longer imaging to needle time, although this did not reach statistical significance, possibly due to the small sample size.

In our institution, patients diagnosed with acute ischemic stroke are directly transferred to computed tomography (CT) scanner without delay. This was reflected in the comparable door to imaging time for both groups in our study. We propose that shifting the main components of acute stroke assessment, including history taking and examination, together with obtaining consent for thrombolysis to the next time interval, i.e., imaging to needle time, was the source of delay in this time interval and ultimately the onset to needle time in patients from culturally and linguistically diverse (CALD) backgrounds.

The similar onset to arrival and door to imaging time intervals also suggest that recognition of stroke symptoms was unlikely to be a contributor.

We also found that CALD patients had longer stay in a stroke unit and were more likely to be discharged to residential aged care facilities independent of age, sex, stroke severity, and comorbidity index. Note the small number of cases (*n* = 7). Other discharge outcomes (in-hospital mortality and discharge to home/rehabilitation) were similar between CALD and patients and non-CALD patients. 

To the best of our knowledge, this is the first report of statistically significant delay in thrombolysis in CALD patients. Rostanski et al. failed to demonstrate an association between language discordance between patient and physician and thrombolysis times [5]. It is important to note that this study was conducted in an American centre with a large proportion of Spanish-speaking patients where in-person Spanish interpreters were available 24 h per day. While in our network, telephone interpreting services are available, access to an in-person interpreter in an Emergency setting is limited and requires prior bookings. The use of interpreter services was not determined in our cohort. 

Shah et al., in a large retrospective study using the registry of the Canadian Stroke Network, showed that patients with language barriers had similar thrombolysis rate and door to needle time but surprisingly less mortality and better quality of care in general compared with those without language barriers [6]. These findings persisted after adjustments for many potential confounders. The authors suggested that reduced mortality in patients with language barriers could be explained by their desire for aggressive care, as shown by the longer length of stay and higher rates of moderate-to-severe residual neurological deficit at discharge [6].

Kilkenny et al., in the only Australian study investigating the impact of language barriers on stroke care, reported more prolonged onset to arrival time (173 min vs. 155 min; *p* = 0.06) in patients who required interpreters, while thrombolysis time interval differences were not reported [8]. Similar to our finding, there was a two-day longer length of stay and higher rates of living with support associated with language barriers [8]. The use of interpreters as an indicator of language barriers expectedly underestimated the true number of patients with language barriers; i.e., language barriers were shown to exist in >7% of strokes in Australia [4] compared to 4.2% who required interpreter in Kilkenny et al. report [8].

Our study has the characteristic limitations of a retrospective study based on medical records. This was a single-centre study that makes the generalisability of the findings uncertain. However, our centre represents an area with a high proportion of migrants in Melbourne, which provides an excellent opportunity for studies into language barriers [9]; i.e., 20% of the study population had a primary language other than English compared to >7% reported nationally [4]. While we conducted an adjusted analysis to a range of potential confounding variables, other potential confounders such as ethnicity and socioeconomic status were not collected. Language disturbance resulting from stroke (aphasia), level of consciousness on presentation, and impact of patients’ premorbid mental health issues, e.g., depression and anxiety, were not accounted for. Similar to other retrospective research into language barriers, the definition of a language barrier used in the study is unlikely to fully capture the patient capacity to communicate effectively and could be a source of over or underestimation. 

## 6. Conclusions

Understanding the impact of linguistic disparities on healthcare in countries such as Australia with ever-growing cultural and linguistic diversity is important. Despite this, only scarce research has been conducted into the impact of language barriers on stroke care and outcome.

Thrombolysis is a time-sensitive therapy where the benefit diminishes with delay [10]. A 15 min delay in thrombolysis is associated with the loss of one -month disability-free life [11]. The presence of a language barrier in our study was associated with an almost 15 min delay in thrombolysis and poorer clinical outcomes after adjustment for several potential confounders. More research is required to investigate the underlying reasons for this disparity and establish methods to improve current practice.

## Figures and Tables

**Table 1:** Study population baseline characteristics.

	English (N = 298)	Non-English (N = 76)	*p*-Value
Age, years, mean (SD)	71.85 (13.58)	76.68 (10.25)	0.003 *
Male (%)	172 (57.72)	40 (52.63)	0.43
Initial NIHSS, median (IQR)	8 (5–15)	9 (6–16)	0.06
In-patient stroke (%)	16 (5)	3 (4)	0.5
Recruitment to clinical trials (%) ^a^	25/115 (21.74)	5/28 (17.86)	0.79
Charlson Comorbidity Index (CCI) (%)			
0	99 (33.3)	22 (28.9)	0.58
1	66 (22.2)	18 (23.7)	0.76
≥2	133 (44.5)	36 (47.4)	0.69
Premorbid modified Rankin Scale (%)			
0	196 (65.77)	35 (46.67)	0.002 *
1	29 (9.73)	14 (18.67)	0.04 *
2	22 (7.38)	12 (16)	0.04 *
3	33 (11.07)	10 (13.33)	0.68
≥4 ^b^	18 (6.04)	4 (5.33)	0.58
Comorbidities (%)			
Hypertension	201 (67.45)	58 (76.32)	0.16
Hyperlipidaemia	124 (41.61)	41 (53.95)	0.06
Diabetes mellitus	87 (29.19)	24 (31.58)	0.67
Ever smoker	79 (26.51)	18 (23.68)	0.66
Ischemic heart disease	81 (27.18)	21 (27.63)	>0.99
Peripheral vascular disease	9 (3.02)	3 (3.95)	0.71
Previous stroke/TIA	56 (18.79)	17 (22.37)	0.51
Atrial fibrillation	73 (24.5)	23 (30.26)	0.30
Renal impairment	30 (10.07)	4 (5.26)	0.26
History of malignancy	45 (15.1)	8 (10.53)	0.36
Cognitive impairment	26 (8.72)	7 (9.21)	0.82
Depression/Anxiety	40 (13.42)	12 (15.79)	0.58

The ^a^ refers to only applicable to 2016 and 2017; ^b^ only one patient from the English group had premorbid mRS of 5; * *p* < 0.05. Abbreviations: National Institutes of Health Stroke Scale, NIHSS; Charlson comorbidity index, CCI; Transient Ischemic Attack, TIA.

**Table 2:** Relevant time intervals by language.

	English	Non-English	Unadjusted Difference [95% CI] ^a^	Unadjusted *p*-Value	Adjusted Difference [95% CI] ^b^	Adjusted *p*-Value
Time intervals, minutes, mean (median, IQR)						
Onset to arrival	85.7 (73.5, 55–104)	87.6 (75, 60–105.5)	1.9 (−8.8–12.8)	0.71	1.3 (−10–12.9)	0.81
Onset to needle	173.4 (167.5, 135–209)	188.5 (180, 155–213)	15.1 (1.3–28.8)	0.03 *	13.7 (0.12–27.4)	0.04 *
Door to imaging	35.8 (27, 16–47)	36.4 (30, 17–50)	0.6 (−5.7–6.9)	0.99	−0.009 (−6.5–6.4)	0.99
Imaging to needle	56.7 (52, 36–73.5)	65.7 (60, 41–77.5)	9 (−0.44–18.4)	0.06	8.9 (−0.57–18.5)	0.06
Door to needle	91.9 (87.5, 65–110)	100.9 (91.5, 73–121.5)	9 (−1.4–19.6)	0.09	8.2 (−2.1–18.5)	0.11

^a^ Univariable Analysis; ^b^ Multivariable Analysis for time intervals: adjusted for age, sex, mRS, NIHSS; * *p* < 0.05; *p*-values calculated with bootstrap with 5000 replications. Abbreviations: CI, confidence interval; mRS, modified Rankin Scale; NIHSS, National Institutes of Health Stroke Scale.

**Table 3:** Clinical outcomes by language.

	English	Non-English	Unadjusted OR (95% CI) ^a^	Unadjusted *p*-Value	Adjusted OR (95% CI) ^b^	Adjusted *p*-Value
Mortality (%)						
In-hospital mortality	34 (11.4)	10 (13.1)	1.1 (0.55–2.5)	0.67	0.89 (0.37–2.1)	0.8
Discharge Destination from Stroke Ward (%) ^c^						
Home	139 (46.6)	29 (38.1)	0.7 (0.42–1.1)	0.18	1 (0.56–1.8)	0.9
Clot retrieval	21 (7)	4 (5.2)	1.1 (0.43–2.9)	0.79	1.1 (0.37–3.3)	0.83
Rehabilitation unit	80 (26.8)	20 (26.3)	0.97 (0.54–1.7)	0.92	0.86 (0.47–1.56)	0.63
Admitted from home discharged to RACF	7 (2.3)	7 (9.2)	4.2 (1.4–12.4)	0.009 *	3.6 (1.1–11.4)	0.02 *
Length of stay ^d^						
LOS under stroke unit, days, median (IQR)	4 (3–8)	6 (3–13)	3.8 ^§^ (0.84–6.9)	0.01 *	3 ^§^ (0.35–5.8)	0.02 *

^a^ Univariable Analysis; ^b^ Multivariable Analysis for clinical outcomes: adjusted for age, sex, CCI, NIHSS; ^c^ Data not shown for patients who were admitted form RACF and discharged to RACF (*n* = 10) or transferred to another hospital for reasons other than clot retrieval (*n* = 13); ^d^ Patients who died or transferred to other hospitals on day zero were excluded. ^§^ Absolute difference with 95% confidence interval; * *p* < 0.05. Abbreviations: Odds Ratio, OR; confidence interval, CI; Residential Aged Care Facility, RACF; length of stay, LOS; Charlson comorbidity index, CCI; National Institutes of Health Stroke Scale, NIHSS.

## Data Availability

Data available from the corresponding author with a reasonable request.
